# Companion Animals’ Roles for AIDS Survivors, Mostly Aging Males, during HIV/AIDS and COVID-19 Pandemics

**DOI:** 10.3390/ani12111449

**Published:** 2022-06-03

**Authors:** Lynette A. Hart, Abigail P. Thigpen, Aubrey H. Fine, Ken Gorczyca, Neil Willits, Raquel Bernaldo, Stefanie Malzyner, Jesús H. Guillén, Katherine D’Amato

**Affiliations:** 1 School of Veterinary Medicine, University of California, Davis, CA 95616, USA; apthigpen@ucdavis.edu; 2 College of Education and Integrative Studies, CA Poly State University, Pomona, CA 91768, USA; ahfine@cpp.edu; 3 Pets Are Wonderful Support/Shanti Project, San Francisco, CA 94109, USA; kcgdvm@gmail.com (K.G.); rbernaldo@shanti.org (R.B.); kdamato@shanti.org (K.D.); 4 Department of Statistics, University of California, Davis, CA 95616, USA; nhwillits@ucdavis.edu; 5 School of Social Welfare, University of California, Berkeley, CA 94720, USA; smalzyner@berkeley.edu; 6 HIV Long-Term Survivors International Network, San Francisco, CA 94102, USA; jesusshaman@yahoo.com

**Keywords:** animal companionship, antiretroviral, cats, disabilities, dogs, isolation, loneliness, resilience, social support, stigma

## Abstract

**Simple Summary:**

Long-term aging survivors of human immunodeficiency virus or acquired immunodeficiency syndrome (HIV/AIDS) were surveyed. Some did or did not have companion dogs or cats while experiencing both the AIDS and COVID-19 (COVID) pandemics. While antiretroviral treatments have reduced AIDS fatalities, survivors still suffer challenges with disabilities and finances. The surveyed 147 HIV/AIDS survivors reported experiencing more frequent stigma, aloneness, and sadness/grief during the AIDS pandemic than during COVID. During AIDS, sadness was greater among those with cats than those without cats. During COVID, older respondents unexpectedly were sad less often than younger ones; dog owners less often felt alone and isolated than those without dogs. Support during AIDS retrospectively was better for older respondents, and better for young gays than young straight ones. During COVID, support was better for men than women. Men with dogs felt more supported than those without; women with pets felt least supported. Compared to dog owners, cat owners more often felt isolated and less often felt supported. Few dog or cat owners received support from family members in either pandemic; but during the AIDS pandemic, dog owners had family support more than cat owners.

**Abstract:**

Long-term HIV/AIDS survivors responded online concerning their experiences during the AIDS and COVID pandemics. Recruited from web-based organizations for AIDS survivors, 147 answered questions on: frequency of experiencing stigma, isolation, aloneness, or grief/sadness; pet ownership; and sources of human support during each pandemic. Conditional inference trees were run to identify relevant demographic factors. Post-hoc comparisons were conducted to compare dog owners and cat owners. AIDS survivors reported more frequent feelings of stigma, aloneness, and sadness/grief during the AIDS pandemic than during COVID. Cat owners’ sadness/grief during AIDS was greater than non-owners. During COVID, older respondents unexpectedly were less often sad/grieving than younger ones; dog owners less often felt alone and isolated than non-dog owners. Support during the AIDS pandemic retrospectively was rated better for older respondents; young gays’ support was greater than young straights. During COVID, support was better for men than women. Contrastingly, women with pets felt less support than those without; men with dogs felt more support than those without. Cat owners more often felt isolated and unsupported during COVID than dog owners. Few dog or cat owners received support from family members in either pandemic; during AIDS, family support was better for owners of dogs than cats.

## 1. Introduction

This paper describes the effects of the COVID-19 (COVID) pandemic and the retrospective effects of the HIV/AIDS (AIDS) pandemic on the lives of long-term survivors of the AIDS pandemic, some of whom had companion animals. These survivors, who are 30% more likely to be infected with COVID than the general population, are defined as people who developed HIV before 1996 [1]. Findings are presented from a national survey of a convenience sample of mostly older male survivors, including the challenges they experienced during both pandemics with stigma, aloneness, isolation, sadness/grief, and lack of social support, and the roles of their companion animals.

### 1.1. Long-Term Survivors of the AIDS Pandemic

The first cases of what would later become known as AIDS were reported in the United States in 1981. More than 1.2 million people still live with AIDS in the United States [2]; about 300,000 of these became infected before 1996, making them long-term survivors. Over half of these long-term survivors are over the age of 50, but some people now in their 20s and 30s acquired the disease at birth or while very young, prior to 1996 [3]. Life expectancy for people living with AIDS and receiving active retroviral therapy has increased significantly from 1996 onward [4]. These long-term survivors were surrounded by people who were dying, and many expected they would die young also. The early AIDS pandemic created psychological burdens with a profound sense of loneliness and isolation [5]. People with AIDS experienced limited social support from their friends and families, often being shunned and stigmatized [6].

### 1.2. Risk Factors Affecting Long-Term AIDS Survivors

The term “AIDS survivor syndrome” was coined in 2012 to describe the psychological results of the HIV epidemic, including anxiety, depression, irritability, lack of future orientation, low self-esteem, substance abuse, social withdrawal, isolation, and survivor’s guilt [7]. Symptoms of this syndrome resemble those of post-traumatic stress disorder (PTSD), as experienced by survivors of natural disasters, violent conflicts, and epidemics. People diagnosed with AIDS in the early 1980s had a life expectancy of less than a year [8], often lost their jobs, and developed numerous disabling conditions, including rare infections, cancers, and mental decline. Some had to relocate or became largely homebound or homeless [9]. Most long-term survivors with AIDS felt their friends and family withdrew; they felt socially shunned, experiencing stigma. Some survivors felt guilty about partners they infected or were angry at someone who endangered them [10]. Many survivors seeing friends die around them were in mourning, and research documented their loneliness and isolation [5,11,12].

Many older survivors with AIDS now live with multiple physical complications from aging and using prolonged active retroviral therapy. These older adults exhibit higher rates of illnesses, including cardiovascular, liver and kidney diseases; cancers; frailty; and osteoporosis [13,14]. HIV may accelerate aging by increased inflammation. Survivors have higher risks of depression and dementia than others their age [14]; many struggle with loneliness and social isolation. These survivors appear at higher risk of severe COVID-19 disease [1]. Older survivors with AIDS are often vulnerable to PTSD [15] and other adverse mental health outcomes, such as depression and dementia, including Alzheimer’s disease [14]. Quality of life decreases with age for them, due to their age-related physical declines and their poorer emotional well-being [16]. These psychological impacts can accumulate and increase their risks of poor mental health and premature death [17]. Yet, an emerging form of social support has been the formation of group connections made on the Internet through social media, as a medium for information and friendship [18].

### 1.3. Dog and Cat Companionship during the Pandemics

Isolation was characteristic for most people with AIDS prior to the retroviral treatment and has been essential for the health of our entire society during the COVID pandemic. People find varied ways of coping with isolation or loneliness. Expansion of electronic communication methods that were employed for teaching, such as Zoom, also provided solutions for loneliness. Some persons focused on the companionship of pets, perhaps also gaining additional social support from people that initially was facilitated by friends’ and neighbors’ interest in their pets, wanting to talk about them or even assist with their care.

Animal companionship generally involves dogs or cats. While people can gain support from their pets, resulting in feeling less lonely, the pets require some care, especially dogs, and this can be challenging for people with AIDS who have disabilities and limited income. Vulnerable populations may especially benefit from being caregivers to animals, as nurturing others can enhance self-esteem and a sense of resilience [19]. Caregivers may feel a sense of autonomy and competence as they build a relationship with their companion animals [20]. Four theories can be used to explain how human-animal interaction can be beneficial to humans: attachment theory, animals acting as social support, the biopsychosocial model, and the biophilia hypothesis [21]. According to the literature on animals acting as a social support and the biopsychosocial model, engaging with animals can decrease social isolation and loneliness and facilitate social interaction with other humans and often dogs [22,23]. Even thinking about cats and dogs can (according to all the theories) provide relief and create a buffer from the effects of social rejection [24]. Finally, the attachment theory emphasizes an animal’s dependence on a human that can promote a sense of competence and the need to be engaged as well as provide nurturance to the human [21].

Pets played an important role in the lives of men with AIDS. By the early 1990s, an estimated 45% of Americans with HIV or AIDS had a pet [25]. Having pets within their homes had a calming effect and lowered the individuals’ stress [26]. Being surrounded with loving pets possibly improved their health and immunity [5]. Men with AIDS who had a close attachment with their pets displayed less depression in comparison to those without pets who seemed more prone to depression and anxiety [25]. Volunteer support systems were created to facilitate petkeeping by people with AIDS, such as Pets Are Wonderful Support program (PAWS) in San Francisco and Pets-DC in Washington DC and other similar programs. Providing instrumental support helped meet the physical and psychosocial needs of people with AIDS; PAWS also educated individuals on procedures for safe petkeeping for those who are immunocompromised [8].

Young and old people, including some in distress, may seek the comfort of companionship. During the society wide COVID pandemic, news stories reported that the number of people adopting pets from shelters increased [27,28]. With owners spending more time at home, dogs that usually were alone at home benefited while limited access to veterinarians and financial hurdles created owner challenges. Sequestering at home impacted how people interacted with their pets. The isolation led to a boom in adoption of dogs that research suggested was an antidote to loneliness [29]. Walking dogs helped people socialize outdoors with other dog owners, suggesting that animals enhanced many people’s quality of life.

In this study, we sought to explore the experiences of long-term survivors with AIDS who have faced serious challenges with their health and wellbeing. We focused on the following questions:

1. How did survivors rate their experienced stigma, aloneness, isolation, sadness/grief, and human support during each of the pandemics?
2. Did age or gender play a role in survivors’ reported experiences?
3. What companion animals did survivors have during each pandemic, and were the animals related to the aloneness, isolation, sadness/grief, and human support that survivors reported?
4. Were there contrasts in experiences among owners of dogs, owners of cats, and non-owners?

## 2. Materials and Methods

A bespoke questionnaire was developed to address the questions relevant to AIDS survivors’ experiences during the AIDS and COVID pandemics. Advised that the survey needed to be short, AIDS survivors, academic professionals, and AIDS service providers teamed up to create the survey, including only 30 questions. In addition to a few demographic items, the questions were created to address the research questions of interest. Members of the team vetted the survey questions among some contacts who were AIDS survivors.

The web-based survey was posted from 1 May until 15 July 2021. The survey was designed to take 10–15 min to complete. A convenience sample was obtained by distributing the survey through Facebook groups serving long-term AIDS survivors and through a targeted listserv specific for AIDS survivors.

Survey participants initially viewed an introduction page inviting them to participate in a survey for long-term survivors of the AIDS pandemic. Participants were required to be at least 18 years of age. Questions addressed their sources of support and experiences with stigma, aloneness, isolation, sadness/grief, and social support during both the early AIDS pandemic (1981–1997) and the current COVID pandemic (2019–2021). A total of 150 survey responses were collected. Of those responses, 147 surveys (117 men, 30 women: 79.6% male) provided sufficient information on whether they had any animal companions or not, and this group was used for analyses. The socio-demographic information gathered included: sex at birth, gender identity, sexual orientation, age, and location, summarized in Table 1. Participants were asked about both HIV and COVID diagnoses; for HIV diagnoses, the approximate date of diagnosis was asked. Participants compared the stigma they experienced during the two pandemics and provided ratings on the extent of their feelings of aloneness, isolation, sadness/grief, and sources of support on a 5-point scale, separately for both the AIDS and COVID pandemics. For example, frequency of feeling isolated was categorized as: never; rarely (monthly); sometimes (weekly); frequently (almost daily); and usually. Additionally, participants were asked questions related to pets, such as: whether they had any pets during the pandemics; positive and negative consequences associated with having a pet(s); and support provided in caring for the pet(s). Occasionally a participant left a question blank.

### Statistical Analyses

Responses were either dichotomous or measured on 5-point Likert scales. As such, normality was unlikely. To assure the lack of normality, Wilk-Shapiro tests were run on each of the Likert scales used in the paper (i.e., what is asked for), and they were all significant with a *p*-value below 0.0001, indicating that the variables are not normal (on their own). Then, statistical results were generated using R, version 3.6.1. The formal comparisons (AIDS versus COVID on a given scale) were all done using Wilcoxon signed rank tests. For these analyses, data were prepared by sorting dog owners who had a dog during the specified pandemic, or not, and similarly with cats. A conditional inference tree [30] was run to identify demographic factors that were associated with the relative stigma experienced during the AIDS pandemic and the COVID pandemic. Additional conditional inference trees were run to identify demographic factors that were associated with a subject’s level of comfort or discomfort regarding isolation, aloneness, and sadness/grief, and the person’s sources of human support during each of the pandemics. Significant differences were often found despite medians shown in the tree diagrams often being no different; thus, means are provided as additional information. Original plots from R are included as figures, with additional enlarged labels added for clarity.

Descriptive statistics with Chi-square and Fisher tests were used to compare reported experiences during each pandemic of people who had dogs with those who had cats.

## 3. Results

Analyses were conducted to assess participants’ overall comparisons of their experiences during the two pandemics. Next, conditional inference trees analyzed for the demographic factors associated with the variables of primary concern: isolation, aloneness, sadness/grief, and sources of support during each pandemic. Finally, data from people who had chosen to keep dogs or cats during each pandemic were compared with each other.

### 3.1. Long-Term Survivors’ Experiences of AIDS versus COVID Pandemics

**Figure 1:**
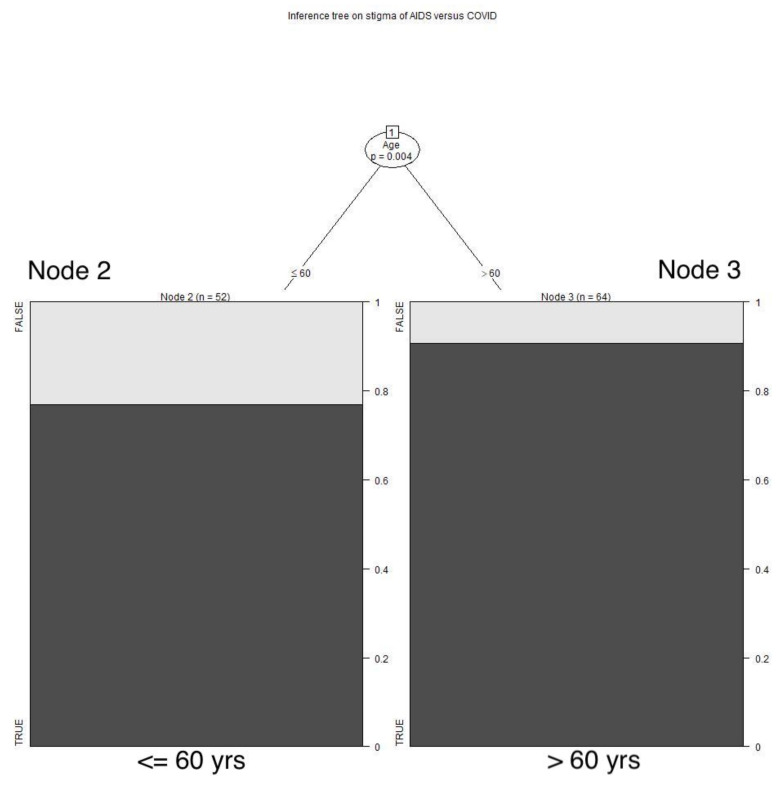
**Stigma of AIDS versus COVID**. Respondents who were more than 60 years of age more often rated the AIDS pandemic as involving significantly more stigma than the COVID pandemic, as compared with younger respondents.

These analyses compared respondents’ recollections of their experiences during the long-term AIDS pandemic with their recent experiences during the shorter COVID pandemic. In terms of experiencing stigma related to having AIDS, 111 respondents rated their experiences during the AIDS pandemic as worse than during the COVID pandemic, 20 rated the two pandemics as similar, and only one said COVID was worse: a highly significant result (*p* < 0.0001). The experience of stigma during the AIDS pandemic was rated as significantly more extreme by the respondents who were more than 60 years of age (*p* = 0.004). See **Figure 1 TREE.**

Using only paired scores, long-term survivors gave higher scores for their frequency of feeling alone during AIDS as compared with during COVID (*p* = 0.00012). Respondents’ frequency of feeling isolated was similar during the two pandemics (*p* = ns), both pandemics involving isolation. Respondents reported having felt sadness and grief more frequently during the AIDS pandemic than during the COVID pandemic, a highly statistically significant result (*p* < 0.0001). Respondents reported the quality of their human support network as better during the COVID pandemic than during the AIDS pandemic (*p* = 0.0016).

### 3.2. Risk Factors Impacting Long-Term Survivors during AIDS and COVID

These analyses examined whether demographic factors such as age, gender, and pet ownership during a pandemic were associated with outcomes such as the frequency of feeling alone, isolated, sadness/grief, or being supported.

**Figure 2:**
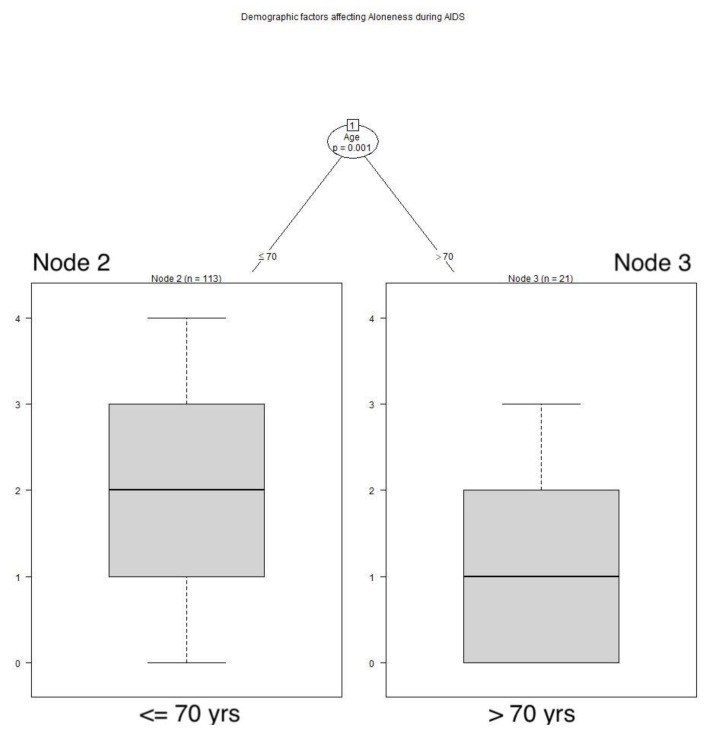
**Demographic factors affecting aloneness during AIDS.** Respondents older than 70 years of age felt alone significantly less often during the AIDS pandemic than younger respondents: Node 2: x^−^ = 1.965, SD 1.1489; Node 3: x^−^ = 1.048, SD 1.0235.

**Figure 3:**
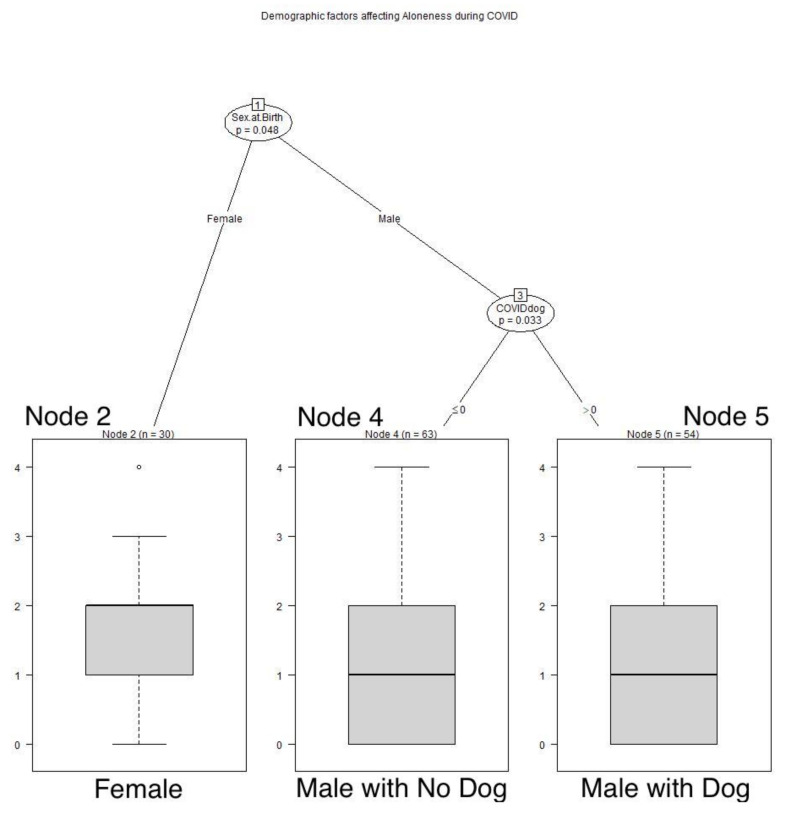
**Demographic factors affecting aloneness during COVID**. Male respondents felt alone less frequently than females, and men with dogs felt alone even less frequently than men without dogs: Node 2: x^−^ = 1.7, SD 1.0554; Node 4: x^−^ = 1.444, SD 1.2152; Node 5: x^−^ = 0.98, SD 1.072.

Age played a role in recollections of feelings of aloneness during the AIDS pandemic. Respondents older than 70 years retrospectively reported feeling alone less often during the AIDS pandemic than younger respondents (*p* < 0.001). See **Figure 2 TREE**. During the COVID pandemic, sex played a role; male respondents felt alone less frequently than females (*p* = 0.048), and the men with dogs felt alone even less frequently than those without dogs (*p* < 0.033). See **Figure 3 TREE**.

**Figure 4:**
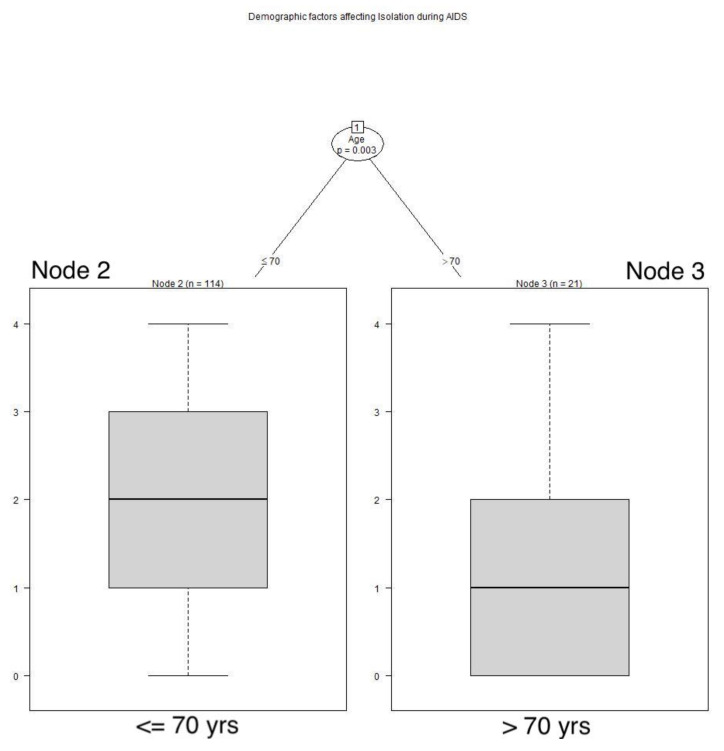
**Demographic factors affecting isolation during AIDS.** Respondents older than 70 years of age felt isolated less often during AIDS than those who were younger: Node 2: x^−^ = 1.912, SD 1.1641; Node 3: x^−^ = 1.048, SD 1.1609.

**Figure 5:**
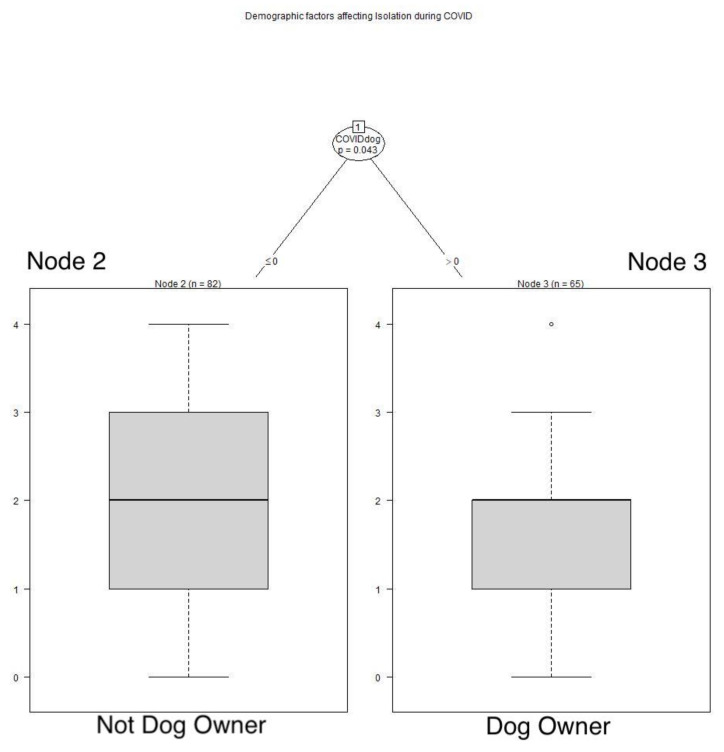
**Demographic factors affecting isolation during COVID.** Respondents with a dog felt isolated less frequently than those without a dog: Node 2: x^−^ = 2.061, SD 1.180; Node 3: x^−^ = 1.677, SD 1.0623.

Similar to feelings of aloneness, age played a role in retrospective feelings of isolation during the AIDS pandemic, with these respondents older than 70 years of age reporting feeling isolated less frequently during AIDS than those younger (*p* = 0.003). See **Figure 4 TREE**. During COVID, those with a dog felt isolated less frequently than those without a dog (*p* = 0.043). See **Figure 5 TREE**. While the group medians for dog owners and non-owners of dogs were the same, the groups differed significantly; the means provide additional information.

**Figure 6:**
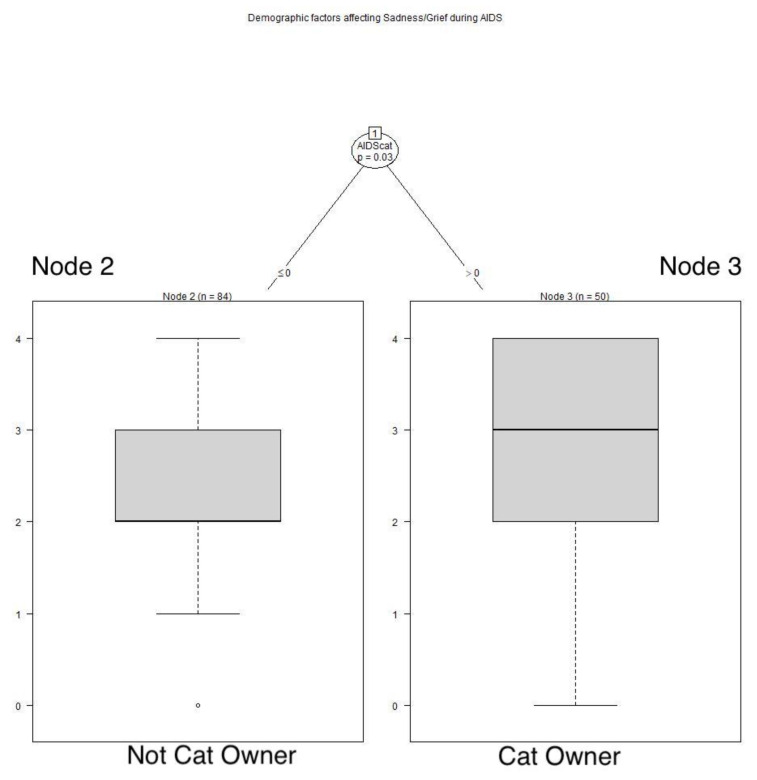
**Demographic factors affecting sadness/grief during AIDS.** Respondents who had a cat during the AIDS pandemic more often felt sadness and grief than those who did not have a cat at that time: Node 2: x^−^ = 2.321, SD 0.9837; Node 3: x^−^ = 2.72, SD 1.0693.

**Figure 7:**
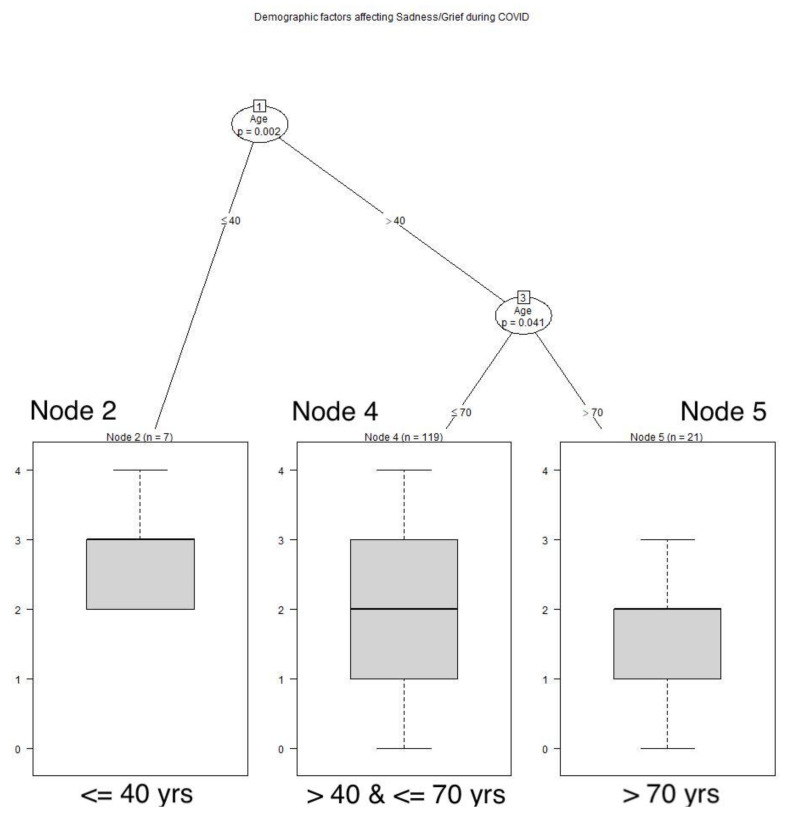
**Demographic factors affecting sadness/grief during COVID.** Respondents 40 years of age or younger felt sadness/grief more often than those who were older; those over 40 years of age through age 70 were sad or grieving more often than those over 70 years of age: Node 2: x^−^ = 2.714, SD 0.7559; Node 4: x^−^ = 1.832, SD 0.9417; Node 5: x^−^ = 1.429, SD 1.0282.

Those who had a cat during the AIDS pandemic retrospectively reported feeling sadness and grief more frequently than those who did not have a cat at that time (*p* = 0.03). See **Figure 6 TREE**. Age played a role during the COVID pandemic, with those 40 years of age or younger being sad more often than older respondents (*p* = 0.002). Those aged over 40 years of age through age 70 years reported having sadness and grief more frequently than those over 70 years of age (*p* = 0.041). See **Figure 7 TREE**.

**Figure 8:**
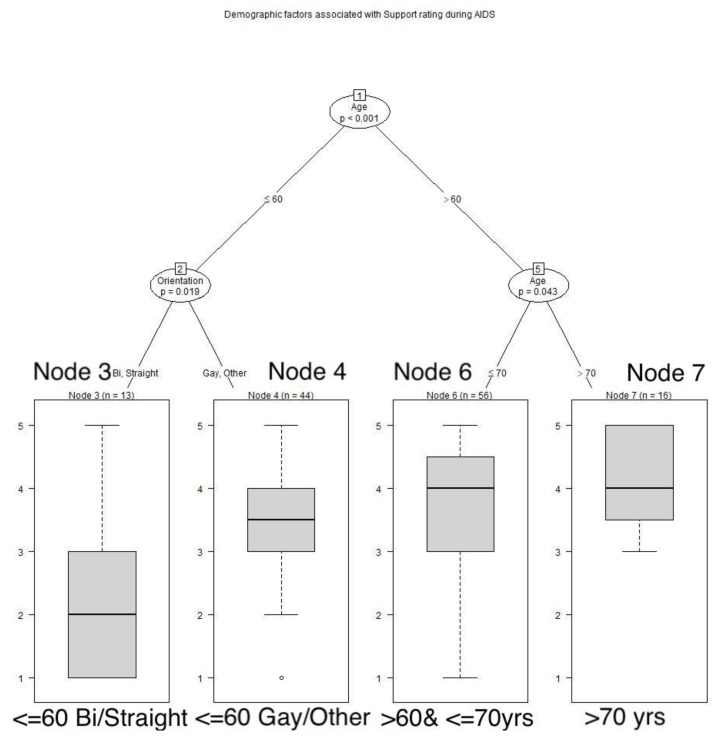
**Demographic factors associated with ratings of human support during AIDS.** Respondents over 60 years of age rated their human support more highly than younger respondents: those over 70 years of age gave even higher ratings; among younger respondents, gays gave higher ratings than straights: Node 3: x^−^ = 2.308, SD 1.2506; Node 4: x^−^ = 3.409, SD 1.272; Node 6: x^−^ = 3.571, SD 1.1258; Node 7: x^−^ = 4.125, SD 0.8062.

**Figure 9:**
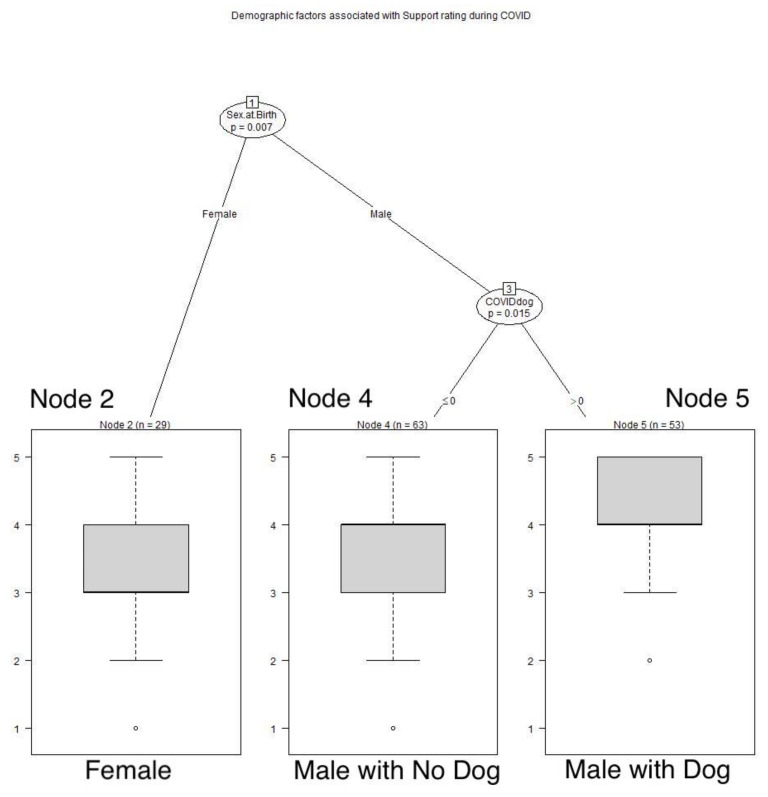
**Demographic factors associated with ratings of human support during COVID.** Men reported greater human support than women, and men with dogs gave still higher ratings of their human support than men without dogs: Node 2: x^−^ = 3.345, SD 0.934; Node 4: x^−^ = 3.683, SD 0.9809; Node 5: x^−^ = 4.094, SD 0.7662.

Age also played a role in recollections of human support that respondents recalled receiving during the AIDS pandemic. These respondents over 60 years of age retrospectively gave better assessments of their human support during the AIDS pandemic than the younger respondents. Among these younger respondents, gays reported having better support during the AIDS pandemic than straights (*p* = 0.019). See **Figure 8 TREE**. Among these respondents over 60 years of age, those older than 70 years reported significantly better human support than the younger group (*p* = 0.043). During COVID, men experienced more adequate human support than women (*p* = 0.007). See **Figure 9 TREE**. Men who had dogs experienced significantly greater human support than those without dogs (*p* = 0.015).

### 3.3. Long-Term Survivors of AIDS with Dogs or Cats during AIDS or COVID

During AIDS but not COVID, a strong majority of cat owners frequently or usually felt alone and sad. People who had ever had a cat described themselves as more stressed during the AIDS pandemic in a composite score of loneliness, isolation, and sadness than those long-term survivors who had ever had a dog (*p* = 0.0007). Among these cat owners, about 26% described themselves retrospectively as usually very stressed in these ways during the AIDS pandemic, as compared with only 9% of these dog owners.

During the COVID pandemic, more than 1/3 of cat owners usually or frequently felt isolated. People who had ever owned a cat felt more isolated than those who had ever had a dog (*p* = 0.0261); this was similar during the AIDS pandemic (*p* = 0.0151). Cumulative high scores on isolation, being lonely, and being sad were higher for those having only a cat during the COVID pandemic, as compared with those having only a dog (*p* < 0.0001). The smaller groups having only either a cat or dog during the AIDS pandemic did not show this significant difference between cat and dog owners.

Similarly, more long-term AIDS survivors who were cat owners felt generally unsupported as compared with dog owners, with the cat owners more often specifying they had no support: this insignificant tendency during the AIDS pandemic (*p* = 0.0670) was significant during the COVID pandemic (*p* = 0.0036). These cat owners significantly more often than the dog owners reported two or more stressors associated with having their cats (*p* < 0.0096). One major stressor for cat owners was transporting their cat to the veterinarians, reported more often by these cat owners than by the dog owners (*p* < 0.015).

Long-term survivors of AIDS who were dog owners found their dogs to have a socializing effect with other people during the AIDS pandemic significantly more than cat owners (*p* < 0.0005); during COVID this was only an insignificant tendency (*p* = 0.0751). Interestingly, during COVID, some dog owners said they received no support at all from their dog, whereas only one cat owner gave this answer about their cat; there was an insignificant tendency toward some dog owners having less support than cat owners from their pets (*p* = 0.0636).

Only a minority of long-term survivors with dogs or cats listed family members as being a source of support during AIDS or COVID, but during AIDS significantly more survivors with dogs than cats had support from family members (*p* = 0.0448). During COVID, there was an insignificant tendency for more long-term survivors with dogs to receive support from a partner or spouse than those with cats (*p* = 0.0657). Most cat owners reported having no human support during COVID. During COVID, analyses of survivors with cats having support from a friend or a spouse as compared with those with dogs having support from a friend or spouse showed that those with cats had less support (*p* = 0.0302); this was not the case during AIDS.

## 4. Discussion

The purpose of this study was to identify the perceptions of long-term survivors who experienced the AIDS pandemic in some past decades and now are experiencing the worldwide COVID pandemic. When exploring the experiences of long-term survivors with AIDS who have faced serious challenges with their health and wellbeing, once again, we focused on the following questions:

1. How did survivors compare their experiences with stigma, aloneness, isolation, sadness/grief, and social support during each of the pandemics?
2. Did age or gender play a role?
3. Were their companion animals related to the survivors’ experiences?
4. How did experiences compare between owners of dogs and owners of cats?

### 4.1. Long-Term Survivors’ Experiences of AIDS versus COVID Pandemics

The harmfulness of stigma for men with AIDS was previously revealed in a study where ratings from 38 gay men with HIV/AIDS were contrasted with those from 278 gay men without HIV/AIDS. Stigma was negatively affected by resilience; those men with HIV/AIDS who had strong resilience had better mental health, and less depression and suicidal ideation, than those with poor resilience [31].

The long-term survivors in this study retrospectively felt their experiences in living through the AIDS pandemic were more challenging to them than their lives experienced during the COVID pandemic; the AIDS pandemic had major impacts for decades and the COVID pandemic for only one to two years at the time of this survey. These respondents reported experiencing greater stigma, aloneness, and sadness/grief during the AIDS pandemic than during COVID, especially the older male respondents, and they reported the quality of human support was worse during AIDS. Not surprisingly, isolation was reported as similar during the two pandemics; society-wide isolation was an aspect of the COVID pandemic.

We are not aware of other research comparing the long-term AIDS survivors experiences during the AIDS and COVID pandemics. Given the huge number of deaths during the AIDS pandemic [14,15], the extreme stigma associated with AIDS [5,32], the long period with a high death rate, and the specific isolation of people suffering AIDS during the AIDS pandemic, one could expect that experiencing the AIDS pandemic at close range while suffering AIDS was much worse than experiencing the COVID pandemic. In contrast, the COVID pandemic brought society-wide isolation and lacked the targeted stigma against people suffering with the specific infection that was characteristic of the AIDS pandemic. The older respondents in this study felt the impact of the AIDS pandemic more keenly; this also could be anticipated as those persons experienced the greater number of deaths occurring in the very early days of the AIDS pandemic.

### 4.2. Risk Factors Impacting Long-Term Survivors during AIDS and COVID

A previous published study found that younger men managed the perceived stigma of AIDS better than older men, perhaps because of the younger men’s stronger support systems, with less stigma and rejection having a positive impact on the younger population [33]. Another study reported that individuals who were HIV positive and aged 50 years and older were more socially isolated than their younger counterparts [34]. Somewhat paradoxically in this current study, among these long-term survivors experiencing their second pandemic, despite their painful extreme experiences and greater stigma during the AIDS pandemic, the older survivors were more prepared with better coping skills for the COVID pandemic, when compared with younger survivors. They suffered less than the younger survivors from aloneness, sadness, and grief; the older ones felt better supported than the younger ones during the COVID pandemic. During COVID, there also was a contrast between men and women, as the men less often felt alone compared with the women.

The older respondents retrospectively experienced the AIDS pandemic as having much greater stigma than the COVID pandemic, and they reported more isolation and sadness/grief plus less support during the AIDS pandemic than the COVID pandemic. Yet, surprisingly, they reported less adverse impacts than younger respondents when remembering the AIDS pandemic, in terms of them feeling isolated or lonely then. During COVID, the male respondents, especially those with dogs, retrospectively did well in less frequently feeling isolated during the current COVID pandemic. Also, during COVID, dog owners felt less alone than non-owners of dogs.

In terms of sadness and grief, younger people fared significantly worse during both pandemics. During the AIDS pandemic, those with a cat felt more sadness and grief than those without a cat. The older respondents also did better in terms of their extent of human support during AIDS. During COVID, women felt less supported than men, especially those with pets; men with dogs felt the most supported. The importance of dogs, specifically for men, stands out.

Apparently, these older survivors, particularly the men who had chosen to acquire dogs, already had better support services in place to continue sustaining themselves and their animal companions. They were prepared to deal with social isolation and cope with the changes during COVID. In contrast, younger respondents seemed to be more blindsided by the COVID pandemic challenges.

The finding that older survivors suffered less stigma, aloneness, and sadness compared to the younger men or to women differs from other studies that have highlighted the extreme vulnerability of AIDS long-term survivors [16,35]. The stigma suffered by people with AIDS is well-documented and those who are older can endure additional ageist exclusion and rejection, feeling more isolated and less supported than younger people with AIDS [36,37]. Respondents in this study differed from these two previous studies, and other similar reports. In recruiting this convenience sample of older AIDS survivors from established web networks, this current study accessed engaged and functioning participants who are in a web community with others facing similar challenges. The somewhat unexpected results of the older men suffering less aloneness and sadness and experiencing better support than younger respondents may reflect the value of participating in the community of long-term survivors: likely selecting for persons with greater efficacy. These persons, particularly men who also had chosen to acquire a dog, scored better on the measures of psychosocial health; their dogs may have added support to their mental health. Other studies have shown socializing effects of having or walking a dog [25,38], and these participants likely made use of these opportunities.

### 4.3. Long-Term Survivors of AIDS with Dogs or Cats during a Pandemic

How do companion animals relate to a person’s mental health, especially during a challenging pandemic? A survey of US pet owners analyzed their reported mental health symptoms prior to and after the COVID pandemic onset, finding that their attachment to pets was a protective factor for those with moderate or high, but not severe, mental health symptoms [39]. Among pet owners in the UK during COVID, animal ownership compared with non-ownership was associated with smaller decreases in mental health for the animal owners and smaller increases in loneliness [40].

When specifically considering people with AIDS, little is known about the specific suitability of cats or dogs. Cats were reported in one study to be comforting and compatible for men with AIDS, offering calming comfort and lacking the greater physical demands that dogs require [41]. Similarly, the psychological health of middle-aged female caregivers of family members with Alzheimer’s disease was supported better among those women with cats than those without cats or with dogs [42]. The lesser demands of the cats seemed a better match for the female caregivers with family responsibilities. Although this current study reveals some challenges faced by the persons who had chosen to acquire cats, in terms of them suffering more aloneness, sadness, and non-support, it also highlights the comfort that all owners received from their cats, whereas this was not always the case with dogs.

Differences reported for personality traits of dog and cat owners include dog people generally being more extroverted, agreeable, and less neurotic than those who identify as cat people [43]. People who chose dogs scored higher on conscientiousness and agreeableness and lower on openness, highlighting differences in those making a choice for cat or dog ownership and human personality. These factors are critical traits in supporting greater mental health. Owners with cats may have a closer relationship with their cats than those with dogs [44]. Women generally are more likely to choose cats as pets than men, indicating that gender contributes to preferring cats [45]. This current study found that men who had chosen to acquire dogs were feeling less alone and sad and better supported than other participants, results that are consistent with them being more extroverted and likely to prefer dogs.

The choice of a cat or dog reflects that cats’ behavior with owners differs from that of dogs. Cats in one study sought a higher level of social contact with their owners, demonstrating the importance cats place on their owners [44]. Cats’ soft fur invites human touch, sometimes at eye level, which further aids intimacy and can offer comfort [46]. Cat’s rubbing against people shows their initiative for contact. At the same time, cats appear more independent than dogs and are more likely to need less physical care from the families they live with and easier to manage. Their independent lifestyle may be an asset in their interactions with persons who are long-term survivors, as noted during interviews [21,38]. Cat owners in our study were unsupported during COVID, but their cats reliably offered contact.

In contrast with cats, dogs offer opportunities for walking and can facilitate socialization with neighbors. During the AIDS pandemic, survivors with dogs in our study more often experienced their dogs facilitating socializations with other people than those with cats. Those with dogs also felt better supported by family members than those with cats. Neither of these was significant during the COVID pandemic, when isolation was world-wide. In some ways, pet ownership seemed more challenging during COVID, with concerns about zoonoses, a care burden that added new problems with shopping or obtaining veterinary care, but perhaps was balanced by better pet welfare due to more human companionship at home [47]. Adoption of pets in the general population increased as the social isolation grew, for example, in an Israel study mentioning dog adoptions as increasing dog walking, happiness, and companionship, combined with less loneliness and depression [29]. Research in India reported that pets helped mediate stress by providing companionship, entertainment, and distraction [48]. A study in Australia described that, during lockdown, dogs protected their owners against loneliness and fostered routine, walks, and socializing, and offered someone to talk to [49]. Everyone experienced various physical, emotional, psychological, and social stressors as they faced mandatory social distancing during COVID, yet a research review showed that even people needing to stay home alone could have some of that stress relieved by a companion animal [50].

Building on the supportive roles that animals can provide, many ongoing programs offer visits with therapy dogs, or provide support to make it easier for people to take care of their own pets. Visits with therapy dogs were curtailed during COVID, leading one program in Canada to convert and develop an online therapy dog program with videos of the dogs and handlers, including mental health and self-care content [51]. About half of these handlers resisted this change, but others stayed with the program and the participants especially enjoyed the online dogs and felt loved and supported by the dogs and connected to them.

Consistent with results of this study, a study in Germany reported that people who were dog owners had higher self-esteem than persons without pets; in particular, male dog owners had higher self-esteem than men without cats or other pets [52]. Further, female cat owners had lower self-esteem than women without pets. In this current study, most cat owners reported having no human support during COVID, and they usually or frequently felt isolated. Also, cat owners more often specified during COVID that they experienced more stressors with their cats than were reported by dog owners. This finding echoes a report from Australia during COVID of cats being a source of worry for their owners [49]. During AIDS, most cat owners frequently or usually felt alone and sad. It seems likely that people who choose to acquire dogs may already differ from those who acquire cats, rather than their animals causing these differences; yet, through the strong socializing effects, acquiring a dog may accelerate enhancing the health of the men who have chosen to get a dog.

Limitations: This was an unusual sample of people in the community of long-term AIDS survivors, including a strong majority of aging male respondents. Recruitment of participants was through web-based groups for long-term AIDS survivors. This procedure likely drew from a pool of persons who are healthier and more engaged with long-term AIDS survivors than the general population of long-term survivors, and certainly more electronically connected. This may partially explain the surprising results of this survey in which older respondents were doing better than younger ones, as compared with other studies, most of which have reported worse outcomes for older men with AIDS.

Among 147 respondents, 28 had not been diagnosed with AIDS (18.9%) but considered themselves deeply involved in the AIDS community and were included in the analyses.

In responding to questions about the two pandemics, survivors were retrospectively considering their memories of the decades-long AIDS pandemic, 20–40 years in their experiences, as well as just 1–2 years of the COVID pandemic at the time of this survey. Naturally the AIDS experience was more extensive and it was to be expected that respondents would rate the AIDS pandemic as worse than the COVID pandemic.

## 5. Conclusions

Older male respondents, despite describing great adversity during the AIDS pandemic, showed current coping that was even further enhanced for those who had chosen to have a dog. The healthier scores of older men regarding their experiences of stigma, aloneness, isolation, and human support were unexpected and differ from other studies of long-term survivors with AIDS. The fact that these respondents belonged to organizations that networked them with other long-term survivors speaks to the importance of community organizing and communication and how it offers meaningful support. The contrasting responses from owners of cats versus dogs suggest that programs providing support in keeping pets to people with special needs could tailor their approaches more specifically for those with cats and dogs: for cat owners to reduce their isolation; for dog owners, assisting with physical care of the dog, such as in walking the dog if the person has mobility challenges.

## Figures and Tables

**Table 1:** Demographics of participants.

Variable/Category	*n*	Percentage
**Gender Identity**		
Female	30	20.41
Queer/non-binary	2	1.36
Male	115	78.23
**Sex at Birth**		
Female	30	20.41
Male	117	79.59
**Orientation**		
Bi	6	4.08
Gay	115	78.23
Other	3	2.04
Straight	23	15.65
**Age**		
18–30	1	0.68
31–40	6	4.08
41–50	8	5.44
51–60	55	37.41
61–70	56	38.10
71–80	18	12.24
81-	3	2.04
**Pandemic_Pet**		
Covid Cat		
No	114	77.55
Yes	33	22.45
Covid Dog		
No	82	55.78
Yes	65	44.22
AIDS Cat		
No	97	65.99
Yes	50	34.01
AIDS Dog		
No	95	64.63
Yes	52	35.37

## Data Availability

The surveys and data presented in this study are openly available in Figshare at https://doi.org/10.6084/m9.figshare.19184375.
